# Mystical but not challenging experiences predict symptom improvement after psilocybin for treatment-resistant OCD

**DOI:** 10.3389/fpsyt.2026.1799543

**Published:** 2026-09-08

**Authors:** Sarah Shnayder, Gabrielle Agin-Liebes, Troy J. Hubert, Terence H.W. Ching, Jamila Hokanson, Christopher Pittenger, Benjamin Kelmendi, Thomas Adams

**Affiliations:** 1 Department of Psychiatry, Yale University School of Medicine, New Haven, CT, United States; 2 Department of Psychology, University of Kentucky, Lexington, KY, United States; 3 Center for Brain and Mind Health, Yale University School of Medicine, New Haven, CT, United States; 4 Yale Child Study Center, Yale University School of Medicine, New Haven, CT, United States; 5 Department of Neuroscience, Yale University School of Medicine, New Haven, CT, United States; 6 Wu-Tsai Institute, Yale University, New Haven, CT, United States

**Keywords:** acute effects, challenging experience, clinical trial, mystical experience, obsessive-compulsive disorder, psilocybin, psychedelic, treatment response

## Abstract

**Background:**

Psilocybin treatment has shown promise across a range of psychiatric conditions. Mystical-type experiences during dosing sessions have been shown to predict the clinical effects of psilocybin treatment in depression, anxiety, and addiction. However, no studies have examined whether acute subjective experiences predict treatment response in obsessive-compulsive disorder (OCD).

**Methods:**

Exploratory analyses were conducted using data from participants with treatment-resistant OCD who received psilocybin as part of a randomized, double-blind, placebo-controlled trial and subsequent open-label phase. Twenty-seven participants who received psilocybin (0.25 mg/kg) completed the Mystical Experience Questionnaire (MEQ-30) and Challenging Experience Questionnaire (CEQ) the day following their session. OCD severity was assessed using the Yale-Brown Obsessive Compulsive Scale (Y-BOCS) at baseline and at 1- and 12-week follow-up.

**Results:**

Greater mystical-type experiences during psilocybin were associated with lower OCD symptom severity at 1- and 12-week follow-up, even after controlling for baseline OCD symptom severity and treatment condition. The Mystical subscale demonstrated the strongest and most consistent associations at both time points, while the Space–Time subscale was only associated with lower Y-BOCS at 12 weeks. The Positive Mood and Ineffability subscales were not significantly associated with post-treatment OCD symptom severity after correction for multiple comparisons. Challenging experiences were not significantly associated with post-treatment OCD severity.

**Conclusions:**

Mystical experiences – particularly experiences of unity, sacredness, and transcendence – during psilocybin sessions are associated with greater OCD symptom reduction. These findings support attention to experiential quality in psilocybin-assisted therapy and have implications for optimizing treatment through dosing, set, setting, and integration practices.

## 1 Introduction

Obsessive–compulsive disorder (OCD) is a chronic and often debilitating psychiatric condition (1). Although first-line treatments, including selective serotonin reuptake inhibitors (SSRIs) and cognitive-behavioral therapy (CBT) with exposure and response prevention, are effective for many patients (2), a significant proportion remain symptomatic despite adequate trial (3, 4), and clinically significant effects typically take months to achieve (5, 6). Novel therapeutic approaches are urgently needed.

In recent years, psilocybin—a naturally occurring psychedelic compound and 5-HT_2_A agonist that can produce profound alterations in perception, cognition, and emotion (7, 8)—has re-emerged as a promising treatment for a range of mental health conditions (9, 10). Psychedelic-assisted therapy commonly involves administering these compounds in controlled settings with structured psychological support, aiming to facilitate meaningful therapeutic experiences (11). Research has demonstrated the therapeutic potential of psilocybin in alleviating depression (12), end-of-life anxiety (13), and substance use disorders (14).

Our group recently completed a randomized, double-blind, placebo-controlled trial of psilocybin with non-directive support for treatment-resistant OCD (15). At the primary endpoint, participants in the psilocybin group demonstrated significantly larger reductions in OCD symptoms than those in the placebo group. However, treatment response was heterogeneous: approximately half of the participants experienced substantial and rapid symptom relief, whereas others showed minimal change. Additionally, a recent randomized clinical trial of repeated psilocybin dosing for OCD similarly found that psilocybin was generally well tolerated and associated with reductions in OCD severity, further supporting clinical interest in psilocybin as a potential intervention for OCD while underscoring the need for larger trials (16). These findings provide preliminary support for the efficacy of psilocybin-assisted therapy in OCD, and – as in the case of established treatments for OCD – highlight marked variability in treatment response. Understanding the sources of this variability is critical for optimizing treatment outcomes.

Emerging evidence suggests that psilocybin may enhance cognitive flexibility and reduce rigid, perseverative thinking patterns characteristic of OCD (17–19). Neuroimaging studies indicate that psilocybin decreases activity and connectivity within the default mode network (DMN) (20, 21), which has been implicated in excessive self-referential processing and rumination in OCD (22–24), Furthermore, the mystical experience—characterized by ego dissolution (25) and a sense of interconnectedness (26)—may directly counteract the intolerance of uncertainty and need for control that are central to OCD psychopathology (27, 28). These considerations provide a theoretical basis for examining whether mystical experiences during psilocybin sessions are associated with therapeutic response in OCD.

Psychedelic sessions at sufficient doses often induce “peak” experiences described as mystical or transcendent—characterized by a sense of unity or oneness, ego dissolution, transcendence of time and space, sacredness, positive mood, and ineffability. Mystical-type experiences have been quantified in the research literature using several self-report instruments, among which the Mystical Experience Questionnaire (MEQ-30) (26) has emerged as the most widely adopted measure. Accumulating evidence indicates that the intensity of mystical experiences during psilocybin sessions is positively associated with clinical improvements across multiple studies and patient populations (29).

Two randomized controlled trials in cancer patients showed that a single high-dose psilocybin session led to rapid and sustained reductions in anxiety and depression, with mystical experience intensity predicting and mediating treatment effects (30, 31). Similar findings have emerged in substance use disorder research. Among smokers receiving psilocybin treatment, those who achieved abstinence at 6 months scored significantly higher on measures of in-session mystical experience than those who continued smoking (32). Another study found that the magnitude of “oceanic boundlessness”—a construct sharing core features with mystical experiences—during psilocybin sessions significantly predicted reductions in depressive symptoms at five-week follow-up (33). Taken together, these findings suggest that a profound mystical-type experience may be a candidate process underlying therapeutic efficacy of psilocybin-assisted therapy.

Psychedelics can also provoke periods of intense fear, confusion, or emotional struggle—commonly described as “bad trips” or challenging experiences rather than uniformly negative or maladaptive states. The Challenging Experience Questionnaire (CEQ) was developed to quantify such difficult aspects (e.g., fear, grief, physical distress, paranoia) of the psychedelic experience (34). The role of challenging experiences in treatment outcome is complex. Some studies and anecdotal accounts indicate that working through challenging experiences can contribute to positive change, with users often later valuing these difficult moments for the insights or emotional breakthroughs that follow (35). In these cases, confronting fear or other negative emotions during the session, especially with proper support, may help resolve psychological conflicts and ultimately improve well-being (35, 36). However, severe challenging experiences also carry risks; they can be overwhelming and, if not skillfully managed, might lead to lasting distress or setbacks rather than therapeutic gain (36). Thus, it remains unclear to what extent challenging psychedelic experiences hinder or support clinical outcomes. This likely depends on factors such as the intensity of the challenge, the individual’s ability to cope or “surrender” to the experience, and the support/integration provided (37).

No published studies have examined the relevance of mystical or challenging experiences during psilocybin in the treatment of OCD. The present study investigated how acute subjective effects of psilocybin relate to subsequent OCD symptom severity. We hypothesized that higher mystical experience scores would be associated with greater symptom reduction, given the literature linking peak experiences to positive outcomes. The role of challenging experiences was explored without a firm *a priori* hypothesis.

## 2 Materials and methods

### 2.1 Participants

The present study is a secondary analysis of data from participants who received psilocybin (N = 28) as part of a phase 2, double-blind, randomized, placebo-controlled trial of psilocybin-assisted therapy for treatment-resistant OCD conducted at Yale University (15). Adults aged 18–65 with treatment-resistant OCD (≥2 failed adequate trials) and Yale-Brown Obsessive-Compulsive Scale (Y-BOCS) scores ≥19 were enrolled; full eligibility criteria and baseline demographics are reported elsewhere (15). One participant was excluded due to missing data at the 1-week follow-up, resulting in a final sample of *N* = 27.

### 2.2 Procedures

Participants were randomized 1:1 to receive a single dose of psilocybin (0.25 mg/kg) or niacin placebo (250 mg) with standardized non-directive psychological support. Participants assigned to niacin were offered open-label psilocybin after completing week 1 assessments; all elected to cross over. Prior to dosing, participants completed two 90-minute preparation sessions. The 6- to 8-hour dosing session was conducted under continuous monitoring by two trained facilitators; participants wore eye shades and listened to a standardized music playlist. Four 90-minute integration sessions were completed post-dosing. Data were pooled across randomized and open-label arms to maximize sample size for these exploratory analyses. Treatment condition (blinded vs. open-label) was included as a covariate in all analyses to statistically control for potential differences between study phases, including expectancy effects associated with unblinded administration.

OCD symptom severity was measured at baseline (1 day prior to dosing, 1 week, and 12 weeks post-dosing. Acute subjective experiences during the dosing session were assessed the day following psilocybin session. Data on OCD symptom severity and acute subjective experiences were compiled from the randomized and open-label study arms.

### 2.3 Measures

Yale-Brown Obsessive-Compulsive Scale (Y-BOCS). The Y-BOCS (38) is a 10-item clinician-administered scale assessing obsession and compulsion severity over the past week. Items are rated 0–4, yielding a total score of 0–40, with higher scores indicating greater severity. The Y-BOCS is the gold-standard measure of OCD symptom severity and was administered at baseline, 1-week, and 12-weeks post-dosing.

Mystical Experience Questionnaire (MEQ-30). The MEQ-30 (26) is a 30-item self-report measure assessing mystical-type experiences occasioned by psychedelics. Items are rated on a 6-point scale (0 = none, not at all; 5 = extreme). The measure yields a total score and four subscale scores: Mystical (15 items; feelings of unity, sacredness, and noetic quality), Positive Mood (6 items), Transcendence of Time and Space (6 items), and Ineffability (3 items). The MEQ-30 was administered the day following the psilocybin session (randomized psilocybin or subsequent open-label psilocybin session for participants initially assigned to placebo).

Challenging Experience Questionnaire (CEQ). The CEQ (34) is a 26-item self-report measure assessing difficult or distressing aspects of psychedelic experiences, including fear, grief, physical distress, insanity, isolation, death, and paranoia. Items are rated on a 6-point scale (0 = none, not at all; 5 = extreme), with higher scores indicating more challenging experiences. The CEQ was administered the day following the psilocybin session (randomized psilocybin or subsequent open-label psilocybin session for participants initially assigned to placebo). CEQ scores were positively associated with ratings of meaning and spiritual significance, constructs closely related to MEQ-30 mystical experience.

### 2.4 Data analytic strategy

All statistical analyses and visualizations were conducted in RStudio (version 4.5.2). Bivariate Pearson correlations were first computed to examine whether baseline OCD severity (Y-BOCS) was associated with the intensity of acute mystical or challenging experiences. Associations between acute subjective experiences during psilocybin administration and OCD symptom severity (1- and 12-weeks post-treatment) were first examined using partial correlation analyses, controlling for baseline Y-BOCS scores and treatment condition (blinded vs. open-label). Partial correlations were computed for MEQ-30 total scores, MEQ-30 subscales, CEQ total scores, and CEQ subscales. Visualizations of significant and null associations were generated using partial regression plots in which both predictor and outcome variables were residualized with respect to baseline Y-BOCS and treatment condition. To account for multiple comparisons, Benjamini–Hochberg false discovery rate (FDR) (39) corrections were applied within each time point across all tested partial correlations. These FDR-corrected partial correlations served as the primary inferential analyses, given the modest sample size and the exploratory nature of examining multiple experiential dimensions.

As a secondary, exploratory analytic step, multiple linear regression models were planned for experiential measures that demonstrated statistically significant associations with post-treatment Y-BOCS outcomes in the primary partial correlation analyses. For measures meeting this criterion, regression models were estimated to examine associations with post-treatment Y-BOCS while controlling for baseline Y-BOCS and treatment condition. These regression analyses were conducted to provide a complementary presentation of the partial correlation results and to facilitate effect size interpretation (noting that standardized effects are equivalent to the corresponding partial correlations), rather than as independent hypothesis tests, Regression models were implemented using JT Tools (40), sjPlot (41), and broom (42) packages.

## 3 Results

Baseline OCD severity was not significantly correlated with mystical experiences (*r* = −.17, *p* = .39) or challenging experiences (*r* = .07, *p* = .74). MEQ-30-Total scores showed significant negative partial correlations with Y-BOCS at both 1-week (*r* = −0.535, *p* <.01) and 12-week follow-ups (*r* = −0.481, *p* <.05; **Table 1A**). Among MEQ-30 subscales, the Mystical subscale demonstrated the strongest associations with post-treatment OCD symptom severity, exhibiting significant negative partial correlations with Y-BOCS at both 1-week (*r* = −0.606, *p* <.01) and 12-weeks (*r* = −0.501, *p* <.05). The Space–Time subscale also showed a significant negative association with Y-BOCS at 12-weeks (*r* = −0.424, *p* <.05). Positive Mood and Ineffability subscales were not significantly associated with 1- or 12-week post-treatment Y-BOCS scores. No CEQ subscales were significantly associated with Y-BOCS at either time point (**Table 1B**). Reported *p*-values reflect Benjamini–Hochberg FDR correction applied within each time point across tested partial correlations.

**Table 1:** Partial Correlations between acute subjective experience measures and post-treatment ocd severity (controlling for baseline severity and treatment condition).

A. Partial correlations between MEQ-30 subscales and Y-BOCS outcomes.										
Variable	Week 1 Y-BOCS	Week 12 Y-BOCS	MEQ-30 total		MEQ-30 mystical		MEQ-30 positive mood		MEQ-30 space time	MEQ-30 ineffability
Week 1 Y-BOCS	1.000									
Week 12 Y-BOCS	0.904***	1.000								
MEQ-30 Total	−0.535**	−0.481*	1.000							
MEQ-30 Mystical	−0.606**	−0.501*	0.936***		1.000					
MEQ-30 Positive Mood	−0.282	−0.235	0.807***		0.630**		1.000			
MEQ-30 Space Time	−0.367†	−0.424*	0.849***		0.676***		0.647**		1.000	
MEQ-30 Ineffability	−0.369†	−0.402†	0.803***		0.662***		0.576**		0.782***	1.000

B. Partial correlations between CEQ subscales and Y-BOCS outcomes.										
Variable	Week 1 Y-BOCS	Week 12 Y-BOCS	CEQ total	CEQ fear	CEQ grief	CEQ physical	CEQ insanity	CEQ isolation	CEQ death	CEQ paranoia
Week 1 Y-BOCS	1.000									
Week 12 Y-BOCS	0.895***	1.000								
CEQ Total	−0.007	−0.155	1.000							
CEQ Fear	0.095	0.069	0.803***	1.000						
CEQ Grief	−0.271	−0.406	0.769***	0.379	1.000					
CEQ Physical	0.177	0.049	0.487†	0.639**	0.031	1.000				
CEQ Insanity	0.053	−0.035	0.604*	0.414	0.287	0.250	1.000			
CEQ Isolation	0.148	0.026	0.638**	0.267	0.604*	−0.108	0.411	1.000		
CEQ Death	−0.138	−0.178	0.149	−0.002	0.252	−0.277	0.110	−0.012	1.000	
CEQ Paranoia	−0.148	−0.228	0.435	0.234	0.244	0.262	0.277	0.398	−0.117	1.000

Partial correlations control for baseline Y-BOCS score and treatment condition. *P*-values were adjusted for multiple comparisons using the Benjamini–Hochberg false discovery rate (FDR) procedure. Values are partial Pearson correlation coefficients (r).*N* = 25 (1A). *N* = 24 (1B). MEQ-30, Mystical Experiences Questionnaire; CEQ, Challenging Experiences Questionnaire; Y-BOCS, Yale-Brown Obsessive Compulsive Scale.

†*p* < .10. *p < .05. **p < .01. ***p < .001.

**Figure 1:**
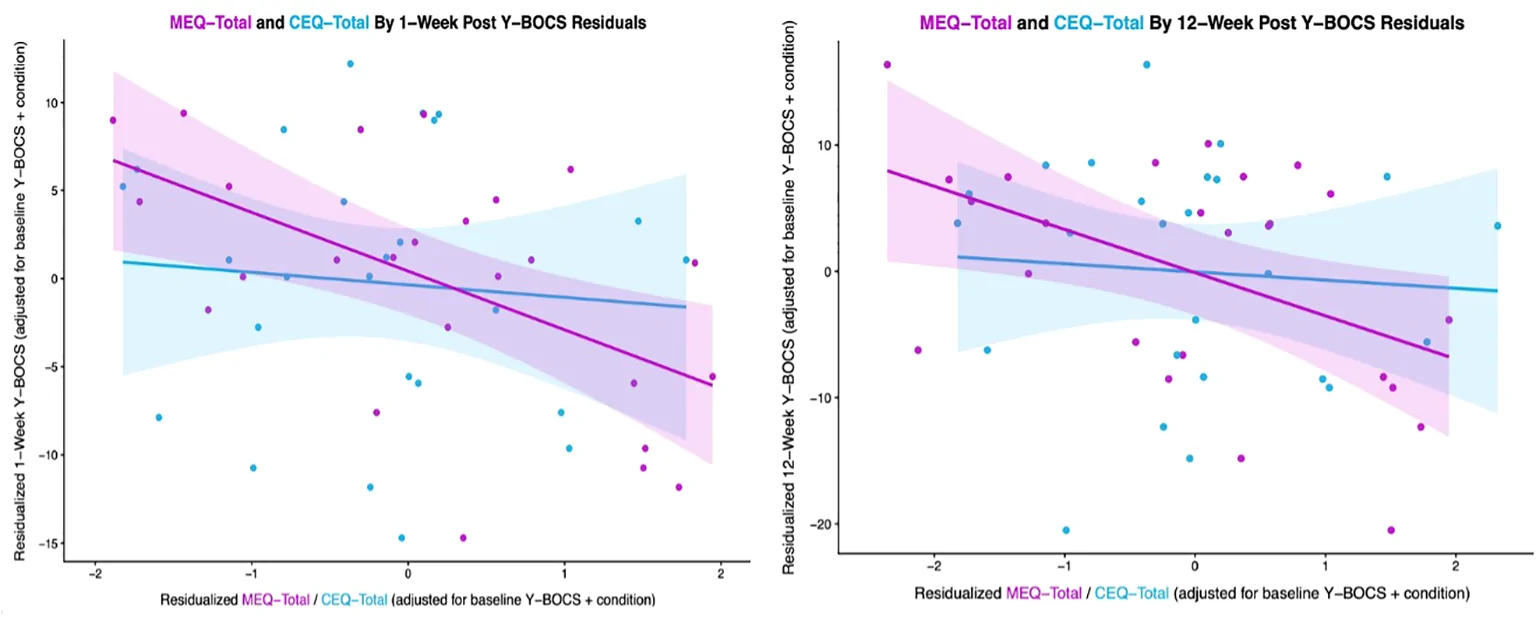
Mystical, but not challenging, experiences were associated with lower Y-BOCS symptom severity at 1-week and 12-week follow-ups. Scatterplots depict partial regression relationships between MEQ-30-Total (purple) and CEQ-Total (blue) scores and residualized post-treatment Y-BOCS scores at 1 week (left) and 12 weeks (right), controlling for baseline Y-BOCS severity and treatment condition (blinded vs. open-label). Higher MEQ-30-Total scores were associated with significantly lower OCD symptom severity at follow-up, whereas CEQ-Total scores were not significantly associated with Y-BOCS outcomes. Shaded bands represent 95% confidence intervals. *Note.* Each line represents a separate partial correlation (not a combined model). Purple = MEQ-30 (Mystical Experiences Questionnaire); Blue = CEQ (Challenging Experiences Questionnaire). Both axes are residualized with respect to baseline Y-BOCS and treatment condition, such that slopes correspond to the partial correlations reported in **Table 1**. MEQ-30, Mystical Experiences Questionnaire; CEQ, Challenging Experiences Questionnaire; Y-BOCS, Yale-Brown Obsessive Compulsive Scale. Both MEQ-30-Total and CEQ-Total scores are mean item scores (0–5 scale).

Challenging experiences were not significantly associated with post-treatment OCD severity. Partial correlations between CEQ-Total and Y-BOCS were near zero at 1-week (*r* = −0.007) and small and non-significant at 12-weeks (*r* = −0.155; **Table 1B**). None of the CEQ subscales showed significant partial correlations with Y-BOCS at either time point following FDR correction. These findings indicate that the intensity of challenging experiences during psilocybin was not related to subsequent OCD symptom outcomes. These findings indicate that the intensity of challenging experiences during psilocybin was not related to subsequent OCD symptom outcomes (**Figure 1**).

## 4 Discussion

Retrospective reports of mystical experiences during psilocybin dosing sessions were prospectively associated with post-treatment OCD symptoms after controlling for baseline OCD symptom severity and treatment condition (randomized vs. open-label). Conversely, challenging experiences during dosing sessions were not significantly associated with post-treatment OCD symptom severity, though this null finding should be interpreted cautiously given the modest sample size. These findings suggest that specific qualitative dimensions of the acute subjective experience—particularly mystical-type features—rather than the intensity of distressing or challenging phenomenology, may be most relevant to therapeutic response in OCD.

When examining MEQ-30 subscales, the largest associations with post-treatment OCD symptom severity were observed for the mystical dimension, which reflects experiences of unity, sacredness, and noetic insight. In contrast, positive mood and ineffability showed weak or non-significant associations with post-treatment OCD symptom severity, whereas the Space–Time subscale demonstrated a moderate association at 12 weeks that did not survive correction for multiple comparisons This pattern mirrors prior psychedelic research indicating subscale-specific predictive validity of the MEQ-30, wherein mystical-type experiences—but not all phenomenological dimensions—are most consistently associated with enduring psychological and behavioral change. Specifically, mystical experiences have been shown to predict perceived spiritual significance and positive life changes, whereas perceptual or temporal alterations alone do not reliably demonstrate such associations (24, 43). It is possible that the mystical factor captures experiences reflecting maximal disruption of self-referential processing, whereas perceptual alterations or temporal distortions involve more intermediate-level changes, dissolution of self-boundaries may represent a psychological process that is uniquely relevant to symptom improvement (35).

One plausible mechanistic account is provided by the REBUS model, which posits that psychedelics weaken entrenched cognitive patterns, facilitating new learning and perspective-taking (44). For OCD patients, the ego dissolution, unity, and insight characteristics of mystical experiences may engage processes that disrupt rigid, overlearned self-referential loops underlying obsessional thinking, offering an experiential demonstration that intrusive thoughts are transient and need not dictate behavior.

The absence of a significant relationship between challenging experiences and post-treatment OCD symptom severity is notable, but should be interpreted with caution, as the study may have been underpowered to detect smaller effects. Prior research varies regarding the potential therapeutic (35, 36) or counter-therapeutic (33) nature of challenging experiences during psilocybin sessions. One interpretation of the null finding is that challenging experiences are not inherently impactful; their therapeutic relevance may depend on whether they are successfully processed, integrated, and imbued with meaning rather than on their intensity alone (33, 45). Commonly used acute experience measures—including the CEQ—index the presence of difficult content but do not directly assess resolution, meaning-making, or successful emotional processing. Some participants with elevated CEQ scores may have struggled without breakthrough, while others may have navigated challenges constructively—gains that would be reflected in their MEQ-30 rather than CEQ scores. This interpretation aligns with research suggesting that acceptance and “surrender” *when navigating* difficult psychedelic experiences, rather than resistance, facilitates emotional breakthrough and positive outcomes (33, 37). Thus, our findings highlight that mystical aspects of the experience are most consistently associated with clinical change, whereas the role of challenging experiences is likely more nuanced and contingent on context, coping strategies, and integration (35, 36). Future research should examine whether the integration of challenging material—rather than its raw intensity—contributes to clinical outcomes.

Qualitative data from a companion interpretative phenomenological analysis (46) provide further context for these findings. Participants frequently described “partial mystical experiences,” with OCD symptoms intruding upon and disrupting full immersion in the psychedelic state. However, those who reported approach behaviors—”letting go” or “surrendering” to arising experiences rather than resisting—described more positive outcomes. Notably, the qualitative analysis revealed that facilitator support was integral for some participants to navigate OCD-related distress during dosing without excessive maladaptive reactions (46). Many individuals with OCD characteristically exhibit a strong need for control and predictability (47), which may render them particularly vulnerable to distress when psychedelic effects intensify.

Several limitations should be acknowledged. The sample size was relatively small (*N* = 27) and racially/ethnically homogeneous, limiting statistical power and generalizability. Expectancy-related influences should also be considered: although part of the study employed randomization and placebo control, approximately half of the sample participated in an open-label psilocybin phase, and expectancy effects were likely present even during blinded conditions given the distinctive subjective effects of psilocybin. Participants may therefore have inferred treatment assignment, introducing potential expectancy effects. In addition, selection effects may have influenced the observed associations, as individuals predisposed to benefit from psilocybin may have been more likely to enroll in the study or to report more intense mystical-type experiences. Other potentially relevant acute processes (e.g., emotional breakthrough or specific insights) were not directly measured and may also contribute to post-treatment outcomes. Finally, follow-up was limited to 12 weeks, and whether these associations persist over longer intervals remains unknown.

In this study of psilocybin-assisted treatment for OCD, mystical-type experiences during dosing were associated with greater reductions in OCD symptom severity, whereas challenging experiences were not. This null finding should be interpreted cautiously given the small sample size and the CEQ’s limited ability to distinguish unresolved distress from difficult experiences that were constructively processed or integrated. Overall, mystical-type phenomenology showed the clearest association with symptom improvement, while the role of challenging experiences may be more context-dependent and nuanced.

## Data Availability

The datasets presented in this article are not readily available due to ethical and privacy considerations related to the small sample size and sensitive clinical content. De-identified data may be made available from the corresponding author upon reasonable request and with appropriate approvals.
